# Compatibility Evaluation of In-Depth Profile Control Agents for Low-Permeability Fractured Reservoirs

**DOI:** 10.3390/gels8090575

**Published:** 2022-09-09

**Authors:** Xuanran Li, Jincai Wang, Jun Ni, Libing Fu, Anzhu Xu, Lun Zhao

**Affiliations:** Research Institute of Petroleum Exploration and Development (RIPED), Beijing 100083, China

**Keywords:** low-permeability fractured reservoirs, nanospheres, single-phase gel particle (PEG), cross-linked bulk gel and swelling particle (CBG-SP), in-depth profile control

## Abstract

Under the background that the in-depth profile control technology is gradually applied in low-permeability fractured reservoirs, this paper regards block H of Changqing Oilfield as the research object, referring to the range of its physical parameters and field application data. Three common in-depth profile control agents (PCAs), nanosphere suspension, poly(ethylene glycol) single-phase gel particle (PEG) and cross-linked bulk gel and swelling particle (CBG-SP), are selected to investigate the compatibility between the fractured channels and the PCAs through a series of experiments. The experimental results show that the nanospheres with particle sizes of 100 nm and 300 nm have good injectivity and deep migration ability, which remains the overall core plugging rate at a high level. The residual resistance coefficient of 800 nm nanospheres decreases in a “cliff” manner along the injection direction due to the formation of blockage in the front section, resulting in a very low plugging rate in the rear section. The injection rate is an important parameter that affects the effect of PEG in the fractured channels. When the injection rate is lower than 0.1 mL/min, the plugging ability will be weakened, and if the injection rate is higher than 0.2 mL/min, the core plugging will occur. The appropriate injection rate will promote the better effect of PEG with the plugging rate > 90%. The average plugging rate of CBG-SP in fractured rock core is about 80%, and the overall control and displacement effect is good. Based on the experimental data of PCAs, the optimization criteria of slug configuration and pro-duction parameters are proposed. According to the principle “blocking, controlling and displacing”, references are provided for PCAs screening and parameters selection of field implementation.

## 1. Introduction

Low-permeability fractured reservoirs have the characteristics of fracture development, low matrix permeability, serious reservoir heterogeneity and anisotropy. During the development process, the injected fluid is prone to cross through the fracture channels and medium or high permeability zones, resulting in low ultimate recovery [1,2]. At the same time, the long-term erosion of the injected fluid makes the physical property and pore structure of the reservoir quite different from the original reservoir characteristics, which affects the later development and application of oil recovery measures [3]. How to improve the phenomenon of high comprehensive water cut in low-permeability fractured reservoirs at the middle and late stage is an urgent production problem to be solved.

Profile control and water plugging technology plays an important role during the development and adjustment of low-permeability fractured reservoirs [3]. The traditional profile control and water plugging methods, such as packer mechanical water plugging, active heavy oil water plugging, cement water plugging, etc. [4], gradually lose the advantages due to its complex treatment process, poor quality controllability, limited action distance and other shortcomings [5,6,7]. In order to better utilize the residual oil in the deep reservoir and achieve the innovation from conventional profile control and water plugging technology to in-depth profile control and displacement, chemical profile control methods stand out from many profile control technologies and become the key measures to reduce water cut and improve production. Based on the action mode of profile control agents (PCAs), chemical profile control methods can be divided into two categories: improve the mobility ratio, or reduce the flow capacity of high-permeability channels. Polymer microspheres and bulk swelling particles commonly used at present belong to the latter.

Considering the application effect of PCAs in low-permeability fractured reservoirs, study on the compatibility between PCAs and channel scale is particularly critical. Shown as Table 1, microsphere suspension, poly(ethylene glycol) single-phase gel particle (PEG) and cross-linked bulk gel and swelling particle (CBG-SP) have been applied to more than 7000 wells in Changqing Oilfield from 2016 to 2018 [8]. Because of their substantial application results and favorable application prospects, they have attracted extensive attention in the domain of research and application of PCAs.

Nanospheres have superior hydration performance and controllable expansion multiple, their excellent profile control effect has led to an increasing trend of related experiments, simulations and field studies on nanospheres. There are two views on the reasons why nanospheres can obtain a good profile control effect. One view is that it depends on the compatibility between the particle size of the expanded nanospheres and the pore throat size of the reservoirs [9], which is mainly based on the three-sphere bridging theory proposed by Barkman J.H. et al. in 1972 [10]. By comparing the relationship between the particle size d and the pore throat diameter D, the plugging situations of the nanospheres are divided into three grades as shown in Table 2. The other view is according to the action mechanism, after hydration and expansion the nanospheres will produce molecular agglomeration phenomenon, and the particle size will be graded. The larger nanospheres will block the large channels through bridging, whereas the smaller nanospheres are easy to migrate to the deep formation and will accumulate or retain in the region with low flow velocity, which increase the specific surface area of the high-permeability layer and reduce the permeability of the layer.

The main function principle of cross-linked bulk gel is to improve the sweep efficiency by increasing the viscosity of displacement system. The research on reservoir adaptability of this PCA mainly focuses on optimizing the concentration of the system and screening the measure wells. When cross-linked bulk gel is used for profile control under reservoir condition, its gel forming time and quality are directly affected by shear dilution, retention adsorption, pH and other factors [11,12]. In addition, it is also a technical problem to be solved that how to maintain the appropriate gel strength so that it can effectively block the dominant channels without completely blocking the formation. In engineering, it is impossible to only rely on cross-linked bulk gel to solve the problem of heterogeneity in the deep formation. This kind of PCA is only suitable for permeability adjustment near the wells [13].

Since Li Yuxiang et al. carried out research on swelling particles in 1999 [14], this kind of PCA has been gradually applied to profile control and water plugging in China. The main mechanism of swelling particles is to accumulate and block at the channels with matching size by water-swelling, and then adjust the subsequent flow direction. For fractured reservoirs, Jiang Haijun et al. found that when the size of particle PCA is greater than 4/5 of the average fracture aperture, it can achieve the effect of bridging plugging [15]. Liu Jialin et al. concluded in their experiments of swelling particles that in order to ensure effective blockage of the dominant channels without completely blocking the formation, the PCAs with particle size less than 4 times the fracture aperture should be selected. The swelling particles are suitable for profile control near the wells in the formation with large pores and fractures [16].

Because single-phase gel particles are newly developed PCAs, there are few relevant research reports at present. We can refer to Bai Baojun’s experimental exploration on similar types of preformed gel particles (PPG) [17]. The experimental results summarize that the migration mode of such particles in the core includes three types: smooth pass, pass after crushing and blockage [18].

Based on previous studies, the advantages and disadvantages of the PCAs that mentioned above are summarized in Table 3.

Although there are many kinds of PCAs, and different PCAs have differences in composition, synthesis method and manufacturing process, there are common uncertainties in the studies of migration and retention law and adaptability of PCAs in various low-permeability reservoirs, which can be divided into three aspects. In terms of geological characteristics, different structural parts have different requirements on blockage strength. For example, large fractures require PCAs with high plugging strength, whereas micro-fracture zone should not inject too much PCAs, otherwise it will lead to production reduction. As for action mechanism, as shown in Table 4, there are contradictions among injectability, plugging intensity and deep migration ability of a certain PCA, and the reasonable combination of various PCAs is one of the ways to solve this problem. In the aspect of agents’ development, the update iteration of PCAs is rapid and the property is uneven, which results in the lack of systematic comparison and demonstration in the analysis of PCAs at present, and it is difficult to be popularized and used for reference.

**Figure 1:**
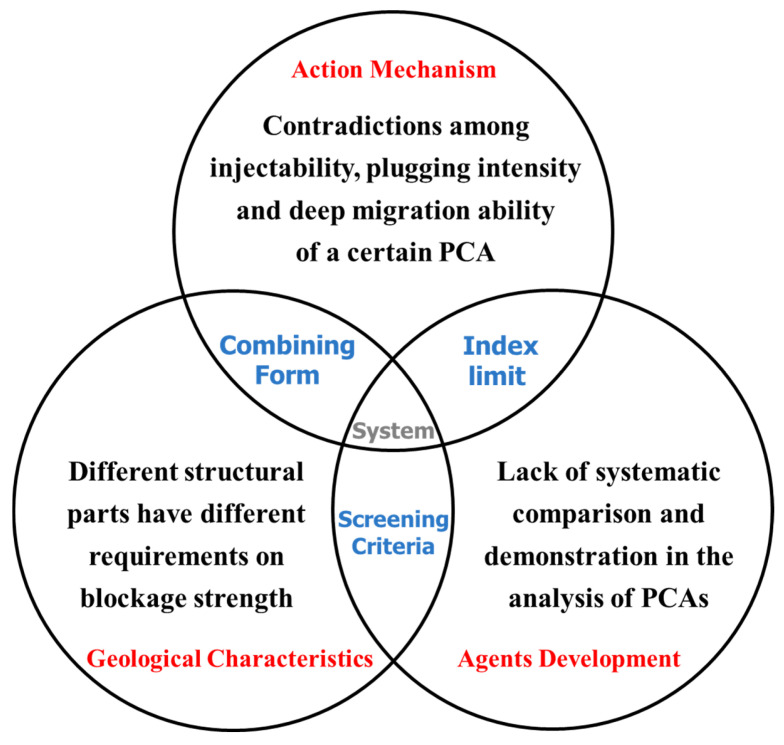
Understanding the compatibility of PCAs and reservoirs from three angles.

With the expansion of implementation scale of the traditional PCAs, the drawbacks have become increasingly prominent. The new prefabricated cross-linked systems arise at the historic moment, but there is still a lack of unified understanding of the compatibility between the new systems and the fracture channels. Therefore, it is imperative to select the PCAs that are suitable for the low-permeability fractured reservoirs, analyze the index limits in Figure 1, and achieve the result of “suit the remedy to the case”.

In this paper, taking Block H of Changqing Oilfield as the research object, the reservoir parameter values are taken as the basis for making artificial cores, and three commonly used PCAs (nanosphere suspension, single-phase gel particle and cross-linked bulk gel and swelling particle) are selected. Based on the field application data, the experimental scheme is designed to study their compatibility with fracture channels.The vertical finite conductivity fracture model is used for well test interpretation. The matrix permeability of the selected artificial core model is about 1 mD and the aperture of fracture channels is 0.2 mm. The optimized 300 mm long core holder equipped with four pressure taps is used to observe the injectability, deep migration ability and overall plugging effect of the PCAs through pressure measurement, and to discuss the influences of important parameters such as injection rate, injection slug and injection concentration, so as to provide a reference for the slug design.

## 2. Results and Discussion

### 2.1. Nanospheres

The particle size of nanospheres is one of the important factors affecting the effect of profile control. The artificial core data and relevant injection parameters (injection volume, injection concentration, injection rate, etc.) selected in this section are listed in Table 5 to explore the differences of plugging ability of nanospheres with different particle sizes in low-permeability fractured reservoirs.

As shown in Table 6, three groups of experiments are designed. Only one level is set for other experimental parameters, except that the particle sizes of nanospheres are set at 100 nm, 300 nm and 800 nm. The injection velocity is 0.3 mL/min, the injection volume is 0.4 PV and the concentration (mass fraction) of the system is 0.4%. The matrix permeability in the artificial core model is about 1 mD, the average diameter is about 0.15–0.17 μm and the aperture of the fracture channel is 0.2 mm. During the experiments, the swelling time of nanospheres is 5 days, and injected into the cores with fractures in a 50 °C thermostat.

**Figure 2:**
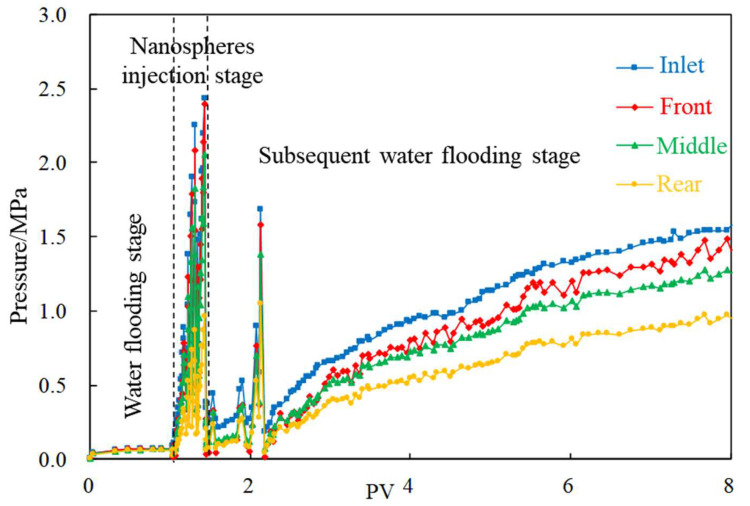
Typical displacement pressure change curve (100 nm nanospheres).

Taking the particle size of 100 nm nanospheres as an example, the migration rule of nanospheres in fracture channel core is described. The following observations can be seen from Figure 2:

(1) Water flooding stage: With the increase in injection volume, the pressures at the four measuring taps along the injection direction begin to rise, and the lifting law of each tap is consistent. The initial pressure increases rapidly, whereas it gradually flattens out in the later stage, and finally stabilizes around 0.1 MPa after a cumulative injection of 1 PV.
(2) Nanospheres injection stage: The pressure at each pressure measuring tap rises rapidly, among which the pressure at the front increases to more than 2 MPa, whereas the pressure at the other pressure measuring taps decreased successively.
(3) Subsequent water flooding stage: The pressure fluctuates to a certain extent, indicating that the flow direction of injected water changes in the core under the influence of nanospheres. Subsequently, the pressure rises steadily. When the water is injected at about 6.5 PV in the subsequent water flooding stage, the front pressure reaches 1.5 MPa, and the pressure drop of each section in the front is approximately equal, whereas the rear pressure drop is higher, reflecting that the nanospheres migrate to the depth of core.

**Figure 3:**
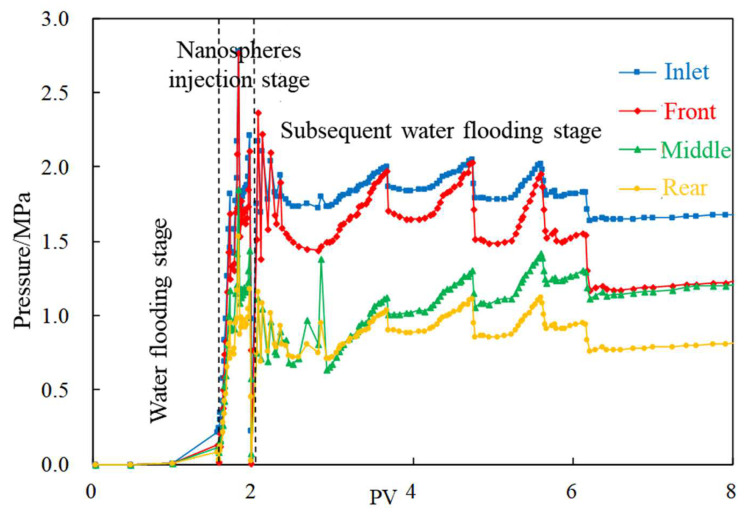
Pressure change curve (300 nm nanospheres).

**Figure 4:**
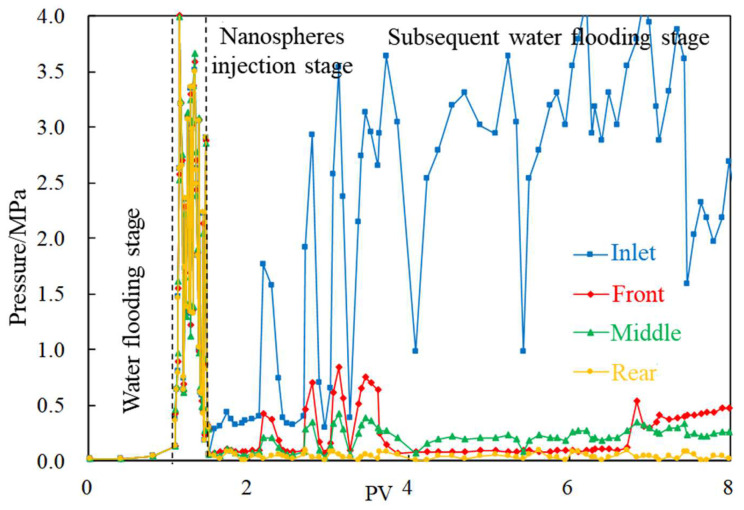
Pressure change curve (800 nm nanospheres).

Experiments A-1~A-3 are conducted to study the influence of nanosphere size. The displacement pressure change curves of 300 nm and 800 nm nanospheres are shown in Figure 3 and Figure 4, respectively. The pressure curve of 300 nm nanospheres at the subsequent water flooding stage maintains a high pressure of 1–2 MPa on the whole, showing a good plugging effect, but with obvious periodic fluctuation. At the same time, the pressure at the inlet end and front begin to decrease significantly after injection of 6 PV, and the pressure difference between them increases, whereas the pressure difference between the middle and rear taps decreases, which means that the main body of the profile control system migrates to the middle.

When nanospheres with particle size of 800 nm are injected, the four pressure change curves all rapidly start up to a high value of 3 MPa. At the subsequent water flooding stage, due to the high pressure at the inlet end, the pressure in the rear section of the core is not significant, which proves that nanospheres mainly stay in the front of the fracture and block the channel, and do not have good migration capacity in the fracture.

Comparing the pressure change curves of experiments A-1~A-3, the calculation results of residual resistance coefficient under the influence of nanospheres with different particle sizes are shown in Table 7. It can be seen that the values of this parameter have a downward trend along the injection direction, in which 800 nm nanospheres have a cliff drop phenomenon, and the profile control effects of the middle and rear sections are worse than that of small-sized nanospheres.

**Figure 5:**
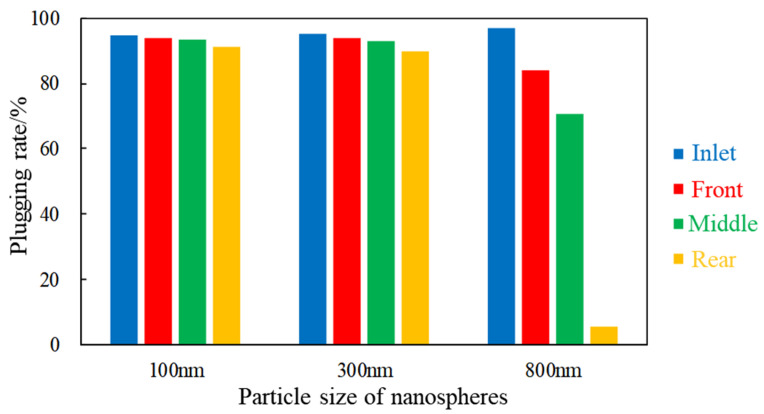
Plugging rate of nanospheres with different sizes at different sections.

Based on the three groups of experimental results, the plugging rates of nanospheres with different particle sizes are calculated, and the calculated values are drawn as the columnar comparison diagram, as shown in Figure 5.

For low-permeability fractured cores, nanospheres with particle sizes of 100 nm and 300 nm have better effect, and the plugging rate at the inlet end remains the highest in the whole interval. Due to the blockage in the front section, the plugging rate of nanospheres with the particle size of 800 nm in the rear section is very low, and these data show that nanospheres with this size do not match the size of the dominant fractured channel.

The above results show that within the size range of the fractured channels, nanospheres with particle sizes of 100 nm and 300 nm can be selected.

### 2.2. Single-Phase Gel Particle (PEG)

Because the single-phase gel particles selected in this paper are made of the fixed formula, and their performance parameters do not fluctuate significantly; thus, the effect of this kind of PCA is not related to its own parameters, and the injection rate becomes the primary factor affecting the profile control effect. The artificial core data and relevant injection parameters (injection volume, injection concentration, injection rate, etc.) selected in this section are listed in Table 8. The purpose of the experiments is to clarify the migration and retention rule of single-phase gel particles.

As shown in Table 9, three groups of experiments are designed. Three levels of injection rate are set at 0.1 mL/min, 0.2 mL/min and 0.3 mL/min, the injection volume is 0.8 PV, the concentration (mass fraction) of the system is 0.5%. The matrix permeability in the artificial core model is 1–3 mD and the aperture of the fracture channel is 0.2 mm. During the experiments, the swelling time of PEG is 15 days, and injected into the cores with fractures in a 50 °C thermostat.

**Figure 6:**
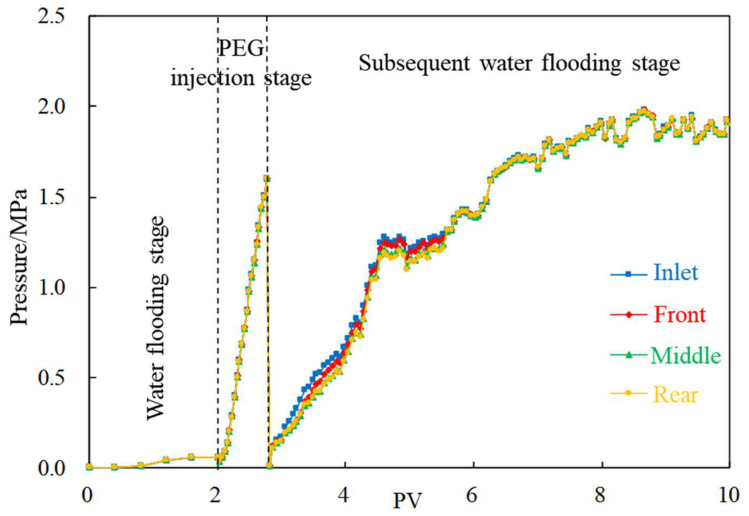
Typical displacement pressure change curve (injection rate is 0.2 mL/min).

Taking the injection rate of 0.2 mL/min as an example, the migration rule of PEG in fracture channel core is described. The following observations can be seen from Figure 6:

(1) Water flooding stage: With the increase in injection volume, the pressures at the four measuring taps along the injection direction began to rise, and the lifting law of each tap is consistent. The initial pressure increases rapidly while gradually flattening out in the later stage, and finally stabilized around 0.1 MPa after cumulative injection of 1 PV.
(2) PEG injection stage: The pressure at each pressure measuring tap rose rapidly, reaching a maximum of more than 1.5 MPa.
(3) Subsequent water flooding stage: The pressure rose steadily after frequent fluctuations, and finally stabilized at about 1.8 MPa.

**Figure 7:**
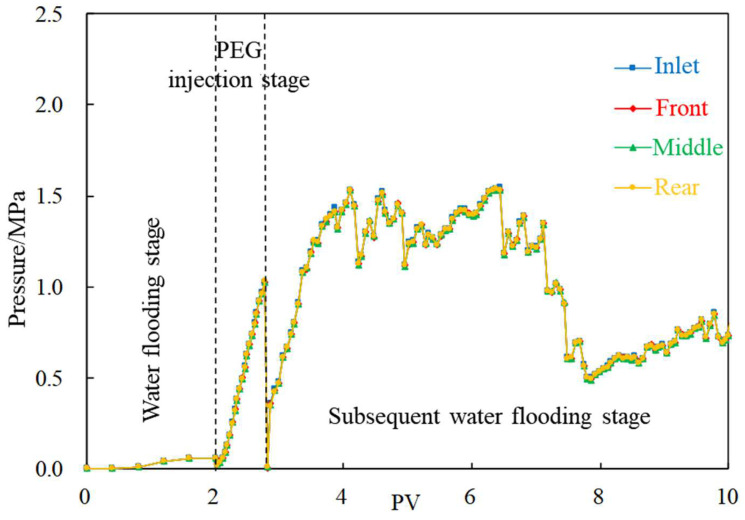
Pressure change curve (injection rate is 0.1 mL/min).

**Figure 8:**
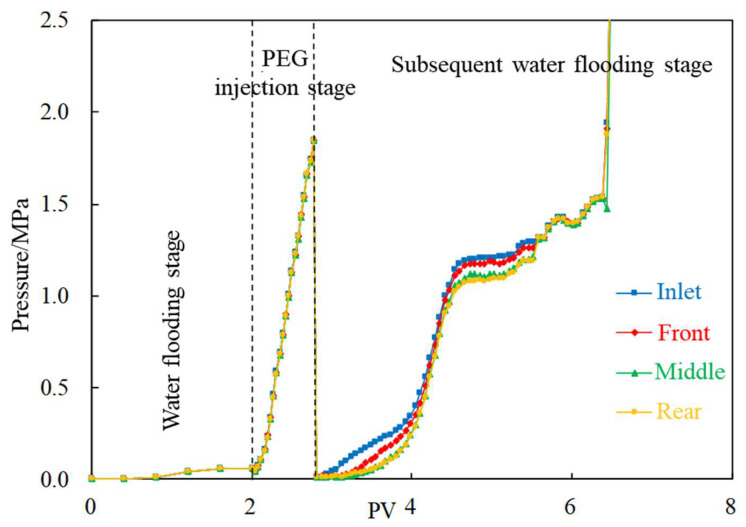
Pressure change curve (injection rate is 0.3 mL/min).

According to experiments B-1~B-3, the pressure curves under different injection rates are drawn, as shown in Figure 7 and Figure 8. In the cores with fracture channels, the pressures of the front, middle and rear sections are basically the same. When the injection rate is 0.1 mL/min, the overall pressure is low, and the plugging effect is ordinary. On the other hand, when the injection rate is 0.2 mL/min, the pressure rises steadily and is generally stable. Therefore, 0.2 mL/min is considered as the optimal injection rate.

**Figure 9:**
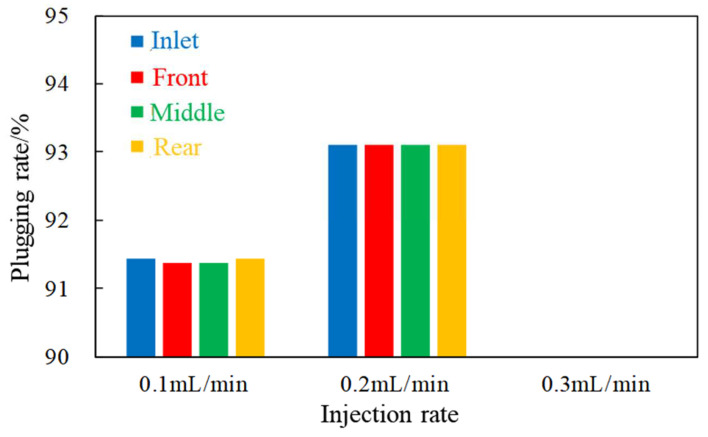
The plugging rates of different positions under different injection rates.

When the injection rate is 0.3 mL/min, the pressure values of each pressure measuring tap are high, and the pressures rise sharply in the later period of the subsequent water flooding stage, even beyond the measurement range, indicating that PEG will cause blockage at this rate. By analyzing the plugging rate statistics of different positions in Figure 9, it shown that a good plugging rate (>90%) can be achieved when the injection rate within the range of 0.1–0.2 mL/min. Blockage occurs when the injection rate is 0.3 mL/min; thus, this rate value is not suitable for this core.

**Figure 10:**
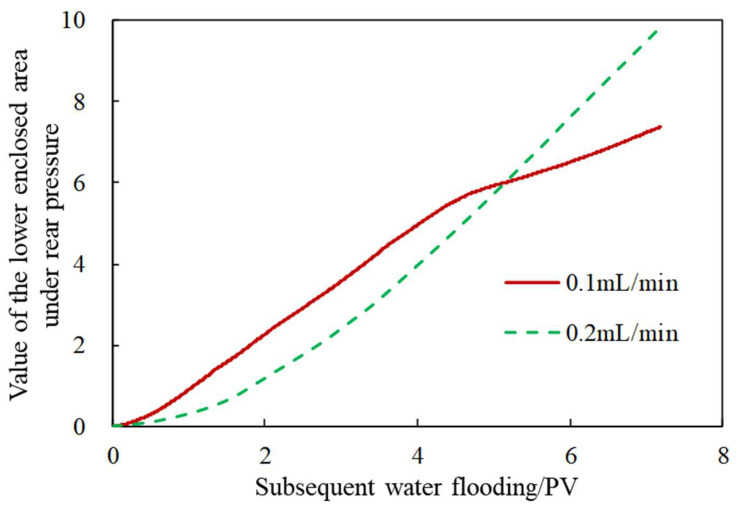
The value of the lower enclosed area under the rear pressure.

Since there is little difference in the plugging rates between 0.1 mL/min and 0.2 mL/min, the rear pressure curves under different injection rates at the subsequent water flooding stage are integrated to obtain the value of the lower enclosed area, as shown in Figure 10. It is found that the pressure integral rises steadily when the injection rate is 0.2 mL/min, indicating that the pressure maintained well at the later stage and the development performance is better under this condition. However, when the injection rate is 0.1 mL/min, the pressure tends to be flat at the later stage, and the pressure holding capacity is relatively poor.

### 2.3. Cross-Linked Bulk Gel and Swelling Particle (CBG-SP)

After years of research and field practice, the slug combination of cross-linked bulk gel and swelling particle and the injection mode with small displacement (2 m^3^/h) have been formed in the process, and the injection volume of a single well is about 1800 m^3^–2200 m^3^. The formula of cross-linked bulk gel is 0.2% polyacrylamide and 0.2% chromium crosslinking agent, whereas the formula of swelling particle is 0.1% polyacrylamide and 0.5% swelling particle. In view of the complete understanding of this kind of PCA, the artificial core data and relevant injection parameters (injection volume, injection concentration, injection rate, etc.) are listed in Table 10, in which the injection parameters are consistent with the field, and this paper only discusses its adaptability in low-permeability fractured reservoirs.

Only one group of experiment is designed as shown in Table 11, the injection volume is 0.5 PV (50%–70% SP + 30%–50% CBG), the injection concentration (mass fraction) is 0.2% HPAM + 0.2% Cr^3+^ + 0.5% SP, and the injection rate is 0.1mL/min. The matrix permeability in the artificial core model is about 3 mD and the aperture of the fracture channel is 0.2 mm. During the experiment, the CBG-SP is injected into the cores with fractures in a 50 °C thermostat.

**Figure 11:**
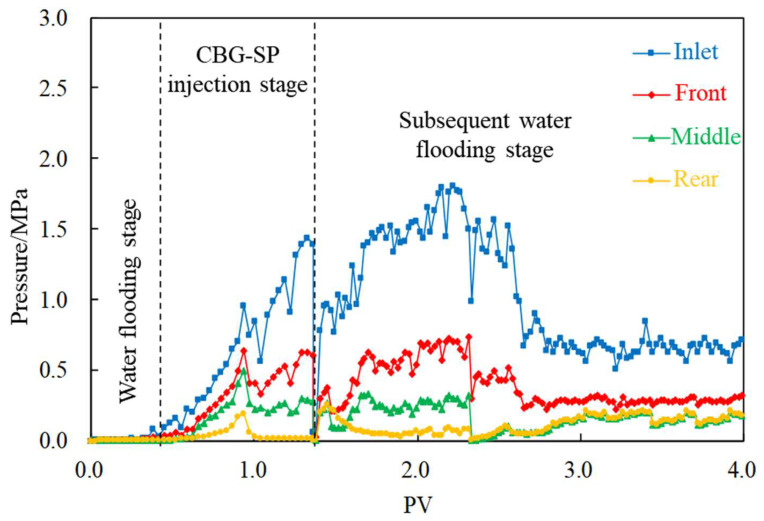
Typical displacement pressure change curve.

Injecting 0.5 PV CBG-SP at the rate of 0.1mL/min into the core with a fracture whose aperture is 0.2 mm to describe its migration rule. The experimental result is shown in Figure 11: The injectability of CBG-SP is relatively good. The pressure rises slightly along the way in the subsequent water flooding stage, and gradually decreases to a lower value and remains stable after injecting water of 1 PV. The plugging rate is between 80% and 90%. Therefore, it is judged that the overall profile control effect of this PCA in the low-permeability fractured core is good.

**Figure 12:**
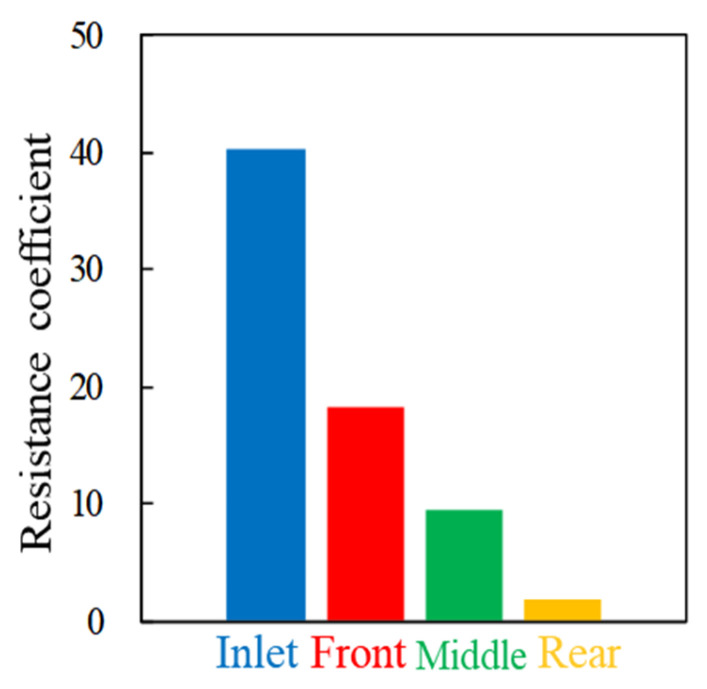
Distribution of the Resistance coefficient at different positions.

**Figure 13:**
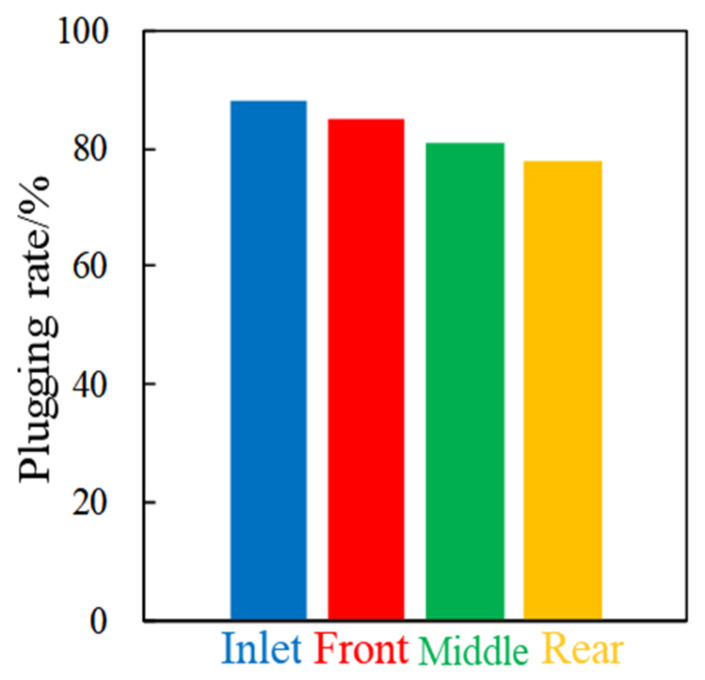
Distribution of Plugging rate at different positions.

According to the calculation results, the distribution diagrams of resistance coefficient and plugging rate under the condition of fracture channel are made, as shown in Figure 12 and Figure 13, respectively. It can be seen from the analysis that the resistance coefficient of CBG-SP is the largest at the inlet end and the smaller at the rear for the fracture channel. From the inlet to the rear, the plugging rate shows a decreasing trend, but the fluctuation is small.

### 2.4. Application Criteria

#### 2.4.1. Type Selection of PCAs

For low-permeability fractured reservoirs, the permeability differences between the inner and outer regions, the dominant channels and the matrix layer are very large, so the permeability difference between each region must be fully considered in the design of profile control schemes, so as to further effectively inhibit water channeling and achieve the goal of improving the effect of increasing oil production and reducing water cut.

For the low-permeability reservoirs with strong heterogeneity, a three-step profile control concept of “blocking, controlling and displacing” can be formed:

(1) After a long period of production and development, the well (inner zone) has a very high permeability value, which can play the high intensity characteristics of CBG, allowing it can play the role of “blocking” near the borehole. SP has good shear resistance and elasticity, which can be used to adjust the water injection profile in borehole zones, so as to take the effect of “controlling”. The slug combination of CBG and SP can be used to block fractures.
(2) In the depth of wells (outer zone), the permeability decreases significantly, and the in-depth displacing is very important. It is necessary to use nanospheres with low viscosity and good injectability to give full play to the advantages of controllable particle size and adjustable expansion multiple, and select appropriate particle size for matching, and migrate to the deep formation step by step for profile control.
(3) As a bridge between inner and outer zones, PEG forms a physical barrier relying on high temperature resistance, salt resistance, good injectability and dispersion, playing the effect of “blocking and controlling”.

According to the above experimental results, the profile control effects of three PCAs for low-permeability fractured reservoirs are summarized, as shown in Table 12.

It can be seen from the above table that for the profile control methods of the fracture channels, PEG or nanospheres with particle size of 100 nm should be preferentially selected, and CBG-SP or nanospheres with particle size of 300 nm should be selected as alternatives.

#### 2.4.2. Parameters Matching

In the field application of in-depth profile control technology, different types of PCAs should be used together as appropriate. For example, polymer, cross-linked agent and swelling particles can be injected together to achieve high-strength gel blocking effect. Using swelling particles with large particle size for slug blocking can protect nanospheres in a deep reservoir. In the slug configuration combination, the three forms shown in Table 13 are recommended: PEG as the main slug, nanosphere as the main slug, and the multi-slug of PEG, CBG-SP and the nanosphere.

For the PCAs that are suitable for the low-permeability fractured reservoirs, combined with field test and application effect, the appropriate parameters are summarized in Table 14:

## 3. Conclusions

In view of the in-depth profile control needs of the low-permeability fractured reservoirs in the late stage of development, this paper uses nanospheres, PEG and CBG-SP to carry out physical simulation experiments, explore the migration and retention rules of different PCAs, clarify the compatibility relationship between PCA types and fracture channels, and form the screening standards of PCA types and slug for the low-permeability fractured reservoirs. The following conclusions are drawn through research and induction:

(1) The experimental results of nanospheres show that the particle size is an important factor affecting the effect of profile control in the low-permeability fractured reservoirs. Among the nanospheres with three different sizes that are selected in this paper, small particles (100 nm) show relatively excellent profile control effect. The reasonable injection parameters in technology are as follows: the injection rate is 0.3 mL/min, the injection volume is 0.4–0.5 PV, the injection concentration is about 0.4%.
(2) The experimental results of PEG show that PEG is suitable to play the role of “blocking and controlling” in the low-permeability fractured reservoirs. The reasonable injection parameters in technology are as follows: the injection rate is 0.2 mL/min, the injection volume is 0.8–1.0 PV, the injection concentration is 0.5%, among which the injection rate has a significant impact on the effect of this kind of agent.
(3) The experimental results of CBG-SP show that this system can achieve the effect of “blocking and displacing” near the well zone in the low-permeability fractured reservoirs. The reasonable injection parameters in technology are as follows: the injection rate is 0.3 mL/min, the injection volume is 1.0 PV, the injection concentration of CBG is 0.2% HPAM + 0.2% Cr^3+^ cross-linked agent, and the injection concentration of SP is 0.1% HPAM + 0.5% SP.
(4) In the implementation of in-depth profile control, according to the actual situation of the well group, the slug form with nanospheres or PEG can be used as the main body, or the synergistic effect of all the four kinds of PCAs that are selected in this paper can be used as the composite slug, which are injected to further improve the effect of increasing oil production and reducing water cut.

## 4. Methodology

### 4.1. Materials

**Figure 14:**
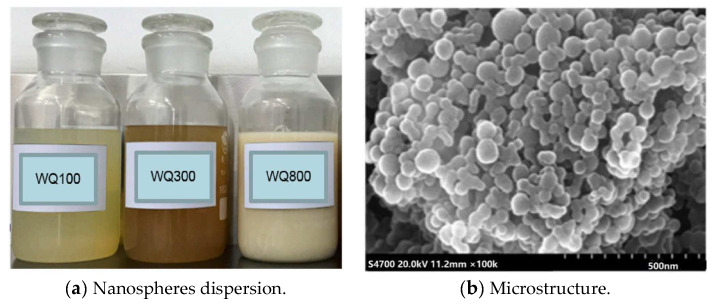
Macro and micro morphology display of nanospheres.

Nanosphere: Nanospheres are polymerized from acrylamide (AM) using functional monomers such as 2-acrylamide-2-methyl-propionic acid (AMPS), n-vinyl pyrrolidone(NVP), and acrylic acid(AA). Figure 14a shows the macromorphology of nanosphere dispersion. When nanospheres are stable in water, they exhibit temperature resistance, salt resistance, and significant mechanical strength. Figure 14b shows the microstructure of nanospheres. Compared with swelling particles, nanospheres have the advantages of small particle size (nanometer to micrometer), low viscosity, easy injection, easier design of expansion time and expansion rate, which are suitable for in-depth profile control [25,26]. In this paper, three kinds of nanospheres with particle sizes of 100 nm, 300 nm and 800 nm are used for compatibility experiments.

**Figure 15:**
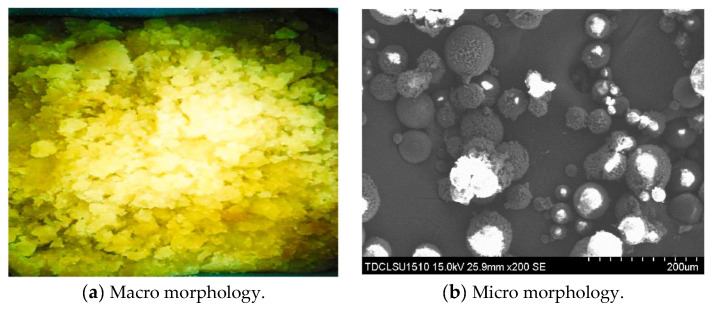
Exhibition of macro and micro morphology of PEG.

Single-phase gel particle (PEG): PEG takes acrylamide, anionic monomer acrylic acid and cross-linked agent as comonomers, and is prefabricated through grinding, granulation and dispersion adopting reverse suspension polymerization. The content of gel in the component is greater than 85%. PEG macroscopically appears as white or light yellow latex, and the particle size is 100–300 μm under scanning electron microscope. Its surface presents the gully-like fold structure. The macro and micro morphologies are shown in Figure 15. PEG has good dispersibility and can withstand high temperatures of 100 °C.

**Figure 16:**
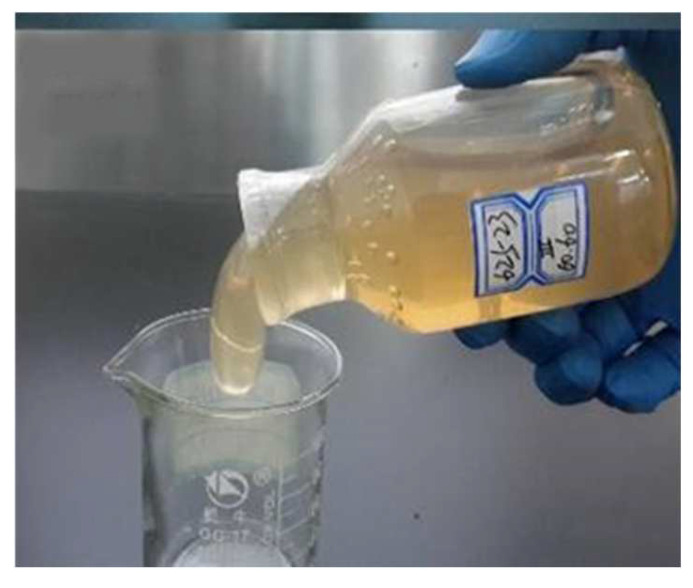
Appearance of CBG.

Cross-linked bulk gel (CBG): In order to overcome the shortcomings such as the ad-sorption film of polyacrylamide aqueous solution is easy to be washed and the effect of reducing the permeability of aqueous phase is unsatisfied, sufficient concentration of cross-linked gel is added to the polymer solution to make the system lose most of its flowing ability and turn into the gel, as shown in Figure 16 [27,28]. Because CBG mainly cross-links after being injected underground, their early fluidity is acceptable. There are differences in the gelling time of commonly used CBG, as short as 5 h or as long as 48 h. After the formation of gel, the strength of CBG increases sharply and the viscosity exceeds 10,000 mPa·s, which has the advantage of strong plugging ability [29]. For the preparation of CBG in this paper, 0.2% polyacrylamide (specific parameters in Table 15), 0.2% Cr^3+^ cross-linked agent, and 0.3% ammonium chloride are used. It has an initial viscosity of 100 mPa·s and is kept in a 50 °C oven.

**Figure 17:**
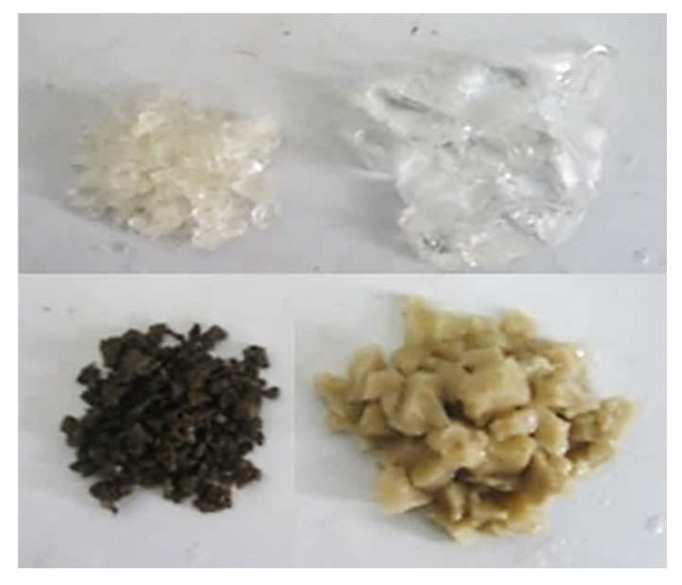
Comparison of SP before and after expansion.

Swelling particle (SP): Swelling particles are made of chemical reagents such as monomers, stabilizers, proppants and swelling agents, which are fabricated through crushing and screening after reaction [30,31,32]. Compared with CBG, SP not only have the advantage of a high plugging rate, but also have the characteristics of controllable gelling conditions, good temperature resistance and salt resistance [33]. In the practice of oilfield, SP can be used as the front slug of cross-linked bulk gel and swelling particle system for profile control, which can be used to treat fractured reservoirs with serious water channeling [34,35]. The SP used in this paper is commercial cross-linked polyacrylamide gel, which was purchased from Zhong Shida Engineering Research Center Co. Ltd. (Beijing, China). After being fully expanded, they exist as dispersed spherical particles at sizes of 3–8 mm. The size changes of SP before and after expansion can be seen in Figure 17. To form a system, the particle gel should be evenly mixed with polyacrylamide solution prior to injection. The concentration of polyacrylamide and SP are 0.1% and 0.5%, respectively. Table 16 shows the SP performance evaluation data.

Synthetic formation water: The total dissolved solid of synthetic formation water is 93,032 mg/L, including K^+^ and Na^+^ 11,832 mg/L, Ca^2+^ 22,289 mg/L, Mg^2+^ 122 mg/L, Ba^2+^ 497 mg/L, Cl^-^ 58,258 mg/L and HCO_3_^−^ 34 mg/L. The density of formation water is about 1.05 kg/m^3^, and the viscosity is 1 mPa·s.

Artificial core: The artificial cores are mainly made of quartz sand, and its length and diameter are 300 mm and 25 mm, respectively.

The above experimental materials and relevant information are shown in Table 17.

### 4.2. Equipment

**Figure 18:**
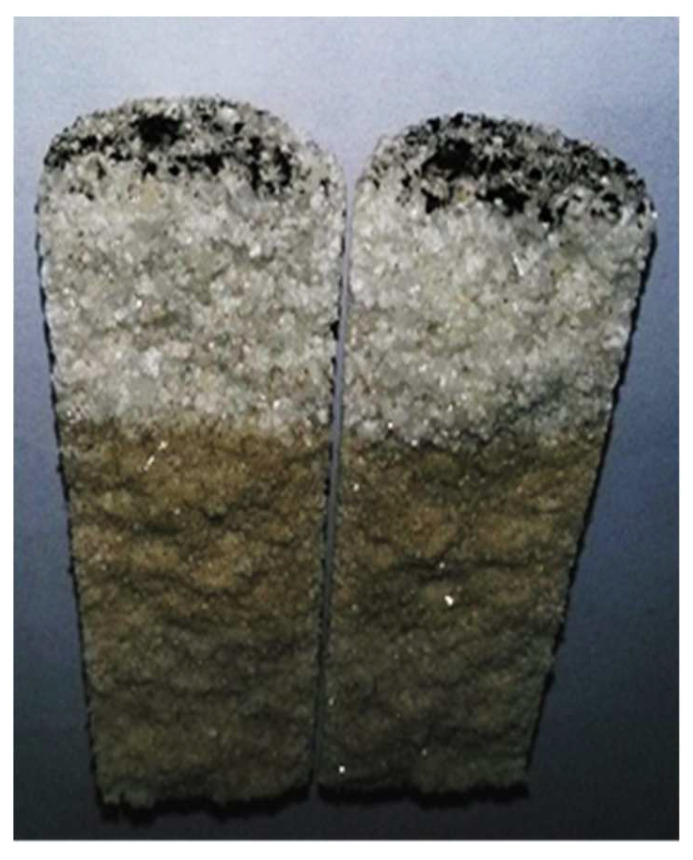
End face accumulation phenomenon.

The equipment and the types used in this experiment are shown in Table 18. Except for special emphasis, all experiments are carried out at a constant temperature of 50 °C. In the pilot test, it was found that due to the small pipe diameter and narrow flow cross-sectional area, the profile control agents in the dispersed phase would accumulate at the inlet end during the injection process, as shown in Figure 18, which would result in abnormal pressure data and led to the failure of the experiments.

**Figure 19:**
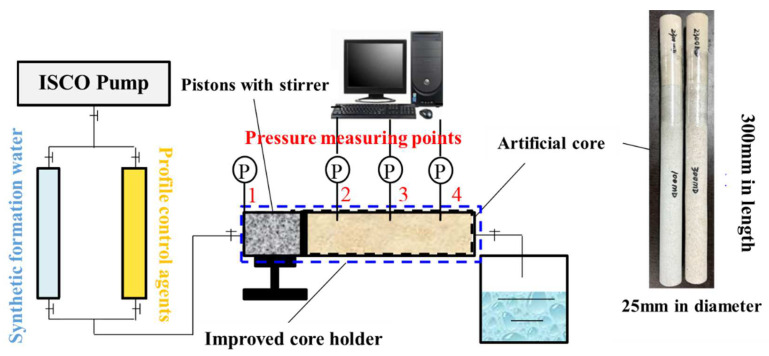
Improved experimental device.

Table 19 records the problems encountered during the experiments and their corresponding solutions. In order to effectively simulate the real injection situation, the original core holder is improved according to the solutions in the table. The improved experimental devices are shown in Figure 19. Four pressure measuring points are set on the improved core holder along the injection direction to study the in-depth profile control capacity and the retention of the PCAs. During the displacement process, the pressure changes at the inlet end (pressure sensor 1), the front section (pressure sensor 2), the middle section (pressure sensor 3) and the rear section (pressure sensor 4) of the cores can be monitored in real time through the data of the pressure measuring points. Among them, the pressure measuring points in the middle and rear sections can provide a reflection of the pressure dynamics of the deep reservoir.

### 4.3. Experimental Procedure

The process of core displacement experiment includes four stages:

(1) Preparation stage: The artificial cores are made according to the permeability values required by the experiments and weighed in advance, install and test experimental devices (pipelines, pressure sensors, etc.). The PCAs are prepared, and the simulated formation water is synthesized.
(2) Water flooding stage: Vacuumize the core and saturate the formation water at a constant rate of 1mL/min. After the displacement pressure difference and the discharge fluid at the outlet are stabilized, the initial water phase permeability is calculated according to Darcy’s Law. The weight of the core is weighed, and the pore volume and porosity of the model are calculated.
(3) PCAs injection stage: PCAs are injected at a constant rate, and the values of each pressure gauge along the path are recorded every 10 s, in which the change of injection pressure with time can be used to investigate its injectability. The confining pressure was 20 Mpa. After injecting about 1 PV, the injection is stopped, and the model is placed in a thermostat to form gel (or expand) at the reservoir temperature. At this stage, the resistance factor Fr can be calculated under a certain velocity:

(1) $$F_{r}=\frac{\Delta p_{si}}{\Delta p_{wi}}=\frac{k_{wi}/\mu_{w}}{k_{si}/\mu_{s}}\left(i=1,2,3,4\right)$$

where $\Delta p_{si}$, $\Delta p_{wi}$ are displacement pressure differences of ith tap during the flow of PCAs and water, respectively; $k_{si}$, $k_{wi}$ are permeabilities of ith pressure tap during the flow of PCAs and water, respectively; $\mu_{s}$, $\mu_{w}$ are viscosities of PCAs and water.

(4) Subsequent water flooding stage:

Reassemble the devices, inject the water at a constant rate of 1mL/min and record the values of the pressure gauges along the path every 10 s. In the process of subsequent water injection of more than 2 PV, the plugging rate $\eta_{i}$ can be calculated by Equation (2):

(2) $$\eta_{i}=\frac{k_{ib}-k_{ia}}{k_{ib}}\times 100\%\left(i=1,2,3,4\right)$$

where $k_{ib}$ is the permeability of ith pressure tap before profile control measures; $k_{ia}$ is the permeability of ith pressure tap after profile control measures.

The average plugging rate can be calculated by Equation (3):

(3) $$\bar{\eta }=\frac{1}{4}\sum_{i=1}^{4}\eta_{i}$$

## Figures and Tables

**Table 1:** Application quantity and effect of PCAs in Changqing Oilfield.

Content	Year			
	2016	2017	2018	2019
Number of wells using microspheres	230	445	3518	3036
Number of wells using PEG	11	100	897	718
Number of wells using CBG-SP	552	714	637	334
The total oil increment/t	109,400	132,200	437,000	380,180
The total water reduction/m³	127,600	150,100	458,200	388,360

**Table 2:** Measurement on the ratio of polymer microspheres to pore throat diameter (according to Wang Tao et al.).

Gradation	Permeability /mD	Average Radius of Pore Throat/μm	Diameter Ratio of Microspheres to Pore Throat	Plugging Efficiency/%
D > 1/3 D	1410	6.11	0.327	33.3
1/3 D < d < 1/7 D	3000	8.94	0.223	26.4
D < 1/7 D	5000	12.00	0.167	25.0

**Table 3:** Summary of the characteristics of the PCAs.

Category	Advantages	Disadvantages	Function
Nanosphere	Good hydration performance; particle size and expansion volume can be adjusted [19]	Poor dispersibility; fragile and easy to break	Displacing in the depth
PEG	Single component, effectively simplify the liquid preparation; small particle size; good dispersibility and injectivity; high resistance to temperature and salt [20,21]	Relatively high cost	Blocking, and controlling at central zone
CBG	Strong blocking capacity after cross-linking; the gel still has an oil displacement effect after shearing	Difficult to control gelation time and gelation quality; multiple components included with complex treatment process; high risk of underground gelation due to differential adsorption; poor salt resistance, easy to hydrolyze, short validity [22,23]	Blocking at near bore zone
SP	Good elasticity; good temperature resistance and shear resistance [16,24]	Poor suspensibility and injectivity; limited migration distance; need to be carried by polymer	Controlling at near bore zone

**Table 4:** Performance differences of different types of PCAs.

Category	Injectability	Plugging Intensity	Deep Migration Ability
Cross-linked bulk gel	Mediocre	Favorable	Mediocre
Swelling particles	Inferior	Favorable	Inferior
Bentonite	Favorable	Inferior	Mediocre

**Table 5:** Influence factors of nanospheres in low-permeability fractured reservoirs.

Profile Control Agent	Reservoir Parameters	Injection Volume, Injection Concentration	Injection Velocity	Nanospheres Size
Nanosphere	Matrix permeability <10 mD; Fracture aperture is about 0.2 mm	0.4 PV, 0.4%	0.3 mL/min	100 nm
				300 nm
				800 nm

**Table 6:** Experimental schemes of nanospheres in low-permeability fractured reservoirs.

Core Number	Porosity/%	Permeability /mD	Fracture Aperture /mm	Research Parameters	Initial Parameters
A-1	12.40	0.89	0.2	100 nm	0.3 mL/min, 0.4 PV, 0.4%
A-2	12.94	1.07	0.2	300 nm	
A-3	12.13	1.20	0.2	800 nm	

**Table 7:** Residual resistance coefficient of nanospheres with different particle sizes.

Stage	Section	Flow Rate /(mL/min)	Pressure Difference /MPa	Residual Resistance Coefficient
Water flooding	Inlet-Rear	1	0.013	-
Subsequent Water flooding (100 nm)	Inlet	0.3	1.614	19.22
	Front	0.3	1.406	16.73
	Middle	0.3	1.268	15.09
	Rear	0.3	0.955	11.37
Subsequent Water flooding (300 nm)	Inlet	0.3	1.724	20.53
	Front	0.3	1.337	15.92
	Middle	0.3	1.169	13.91
	Rear	0.3	0.835	9.94
Subsequent Water flooding (800 nm)	Inlet	0.3	2.879	34.28
	Front	0.3	0.522	6.21
	Middle	0.3	0.288	3.43
	Rear	0.3	0.089	1.06

**Table 8:** Influence factors of PEG in low-permeability fractured reservoirs.

Profile Control Agent	Reservoir Parameters	Injection Volume	Injection Concentration	Injection Velocity
PEG	Matrix permeability <10 mD; Fracture aperture is about 0.2 mm	0.8 PV	0.5%	0.1 mL/min
				0.2 mL/min
				0.3 mL/min

**Table 9:** Experimental schemes of PEG in low-permeability fractured reservoirs.

Core Number	Porosity/%	Permeability /mD	Fracture Aperture /mm	Research Parameters	Initial Parameters
B-1	11.47	1.39	0.2	0.1 mL/min	0.8 PV, 0.5%
B-2	12.02	1.55	0.2	0.2 mL/min	
B-3	12.15	2.28	0.2	0.3 mL/min	

**Table 10:** Influence factors of CBG-SP in low-permeability fractured reservoirs.

Profile Control Agent	Reservoir Parameters	Injection Volume	Injection Concentration	Injection Velocity
CBG-SP	Matrix permeability < 10 mD; Fracture aperture is about 0.2 mm	0.5 PV (50%–70% SP + 30%–50% CBG)	0.2% HPAM + 0.2% Cr^3+^ + 0.5% SP	0.1 mL/min

**Table 11:** Experimental scheme of CBG-SP in low-permeability fractured reservoirs.

Core Number	Porosity/%	Permeability /mD	Fracture Aperture /mm	Research Parameters	Initial Parameters
C-1	13.41	3.05	0.2	0.1 mL/min	0.5 PV, 0.5%

**Table 12:** Comparison between the effects of different PCAs.

Type		Effect of PCA in the Low-Permeability Fractured Reservoir
Nanospheres	100 nm	Excellent
	300 nm	Favorable
	800 nm	Mediocre
PEG		Excellent
CBG-SP		Favorable

Note: Excellent: when average plugging rate > 90%; Favorable: when average plugging rate ranges from 80% to 90%; Mediocre: when average plugging rate ranges from 60% to 80%; Inferior: when average plugging rate < 60%.

**Table 13:** Three practical slug configurations of PCAs.

Slug I	Contents	Slug II	Contents	Slug III	Contents
Pre-slug	Water injection	Pre-slug	Water injection	Pre-slug	PEG
Main slug	PEG	Main slug	100 nm nanosphere	Main slug	CBG-SP
Displacement slug	Displacement (50 m^3^)	Displacement slug	Displacement (100 m^3^)	Displacement slug	100 nm nanosphere

**Table 14:** Suitable parameters of PCAs in low-permeability fractured reservoirs.

Type	Appropriate Injection Volume	Appropriate Injection Concentration	Appropriate Injection Rate
100 nm nanosphere	0.4~0.5 PV	0.4%	0.3 mL/min
PEG	0.8~1 PV	0.5%	0.1–0.2 mL/min
CBG-SP	0.5 PV	0.5%	0.1 mL/min

Note: CBG-SP: The optimal injection concentration of CBG is 0.2% HPAM + 0.2% Cr^3+^ cross-linked agent, and the appropriate injection concentration of SP is 0.1% HPAM + 0.5% SP.

**Table 15:** Polyacrylamide parameters in CBG.

Parameters	Value
Dissolution Time/h	≤2
Solid Content/%	≥88
Hydrolysis Degree/%	20–30
Viscosity-average Molecular Weight/×10^6^	≥17.0

**Table 16:** Performance evaluation data of SP.

Performance Parameters	Value
Solid Content/%	≥30
Mass Expansion Multiple	7–20
Compressive Strength/N	≥500
Salt Resistance (Mass Expansion Multiple in Synthetic Formation Water)	5–10
Temperature Resistance (Strength retention after immersion in simulated formation water under formation temperature for 6 months)/%	≥80

**Table 17:** Information on experimental materials.

Material Name	Types
Nanospheres dispersion	The particle size includes 100 nm, 300 nm and 800 nm The solid content is 20%
Single-phase gel particle (PEG)	Gel content ≥ 85.0%
Polyacrylamide	Solid content ≥ 88%
Chromium cross-linked agent	Cr^3+^ content ≥ 3.0%
Swelling particle (SP)	Solid content ≥ 30%
Deionized water	Provided by the laboratory of College of petroleum engineering, China University of Petroleum (Beijing, China)
Sodium Chloride (NaCl)	AR, Produced by Beijing Yili Fine Chemicals Co., Ltd. (Beijing, China)
Sodium Carbonate (Na_2_CO_3_)	AR, Produced by Modern Oriental (Beijing) Technology Development Co., Ltd. (Beijing, China)
Calcium Chloride (CaCl_2_)	AR, Produced by Modern Oriental (Beijing) Technology Development Co., Ltd. (Beijing, China)
Artificial core	Laboratory of College of Petroleum Engineering, China University of Petroleum (Beijing, China)

**Table 18:** Information on experimental equipment.

Equipment Name	Types
Thermostat	Jiangsu Hai’an Huada Petroleum Instrument Factory
electrothermal blowing dry box	Jiangsu Hai’an Huada Petroleum Instrument Factory
ISCO pump	Jiangsu Hai’an Huada Petroleum Instrument Factory
Electric magnetic stirrer	Jiangsu Hai’an Huada Petroleum Instrument Factory
High pressure core holder	TY-4: Length 300 mm, Diameter 25 mm
Constant-flux pump	2PB00D, Range 0~10 mL/min, 40 MPa
Intermediate container, electronic balance, six-way valve, vernier caliper, beaker, measuring cylinder, glass rod, spoon, dropper, weighing paper, etc.	

**Table 19:** Problems and solutions in the experiments.

Number	Problems	Solutions
1	PCAs are easy to sink when injected under static conditions.	Use pistons with stirrer.
2	PCAs are easy to block the pipeline and lead to abnormal injection.	Use threaded pipeline with inner diameter of 6 mm.
3	Conventional short cores cannot fully study the migration ability of PCAs.	Use 300 mm long core and holder.

## Data Availability

Data available on request from the authors.
